# Southern Europe is becoming climatically favourable for African birds: anticipating the establishment of a new species

**DOI:** 10.1186/s12983-023-00496-x

**Published:** 2023-05-17

**Authors:** Sandro López-Ramírez, Darío Chamorro, Raimundo Real, Antonio-Román Muñoz

**Affiliations:** Biogeography, Diversity, and Conservation Research Team, Department of Animal Biology, Faculty of Sciences, University of Malaga, Malaga, Spain

**Keywords:** African birds, Climate change, *Emberiza sahari*, Favourability, Strait of Gibraltar

## Abstract

**Background:**

The current modification of species distribution ranges, as a response to a warmer climate, constitutes an interesting line of work and a recent challenge for biogeography. This study aimed to determine if the climatic conditions of southern Europe are adequate to host a typical African species, the House Bunting, which is registered regularly during the last years, still in low numbers. To this end, the distribution of the species in its native range was modelled, both in the present and in future climate scenarios, using its current breeding distribution areas and a set of environmental variables.

**Results:**

The results showed that the southern half of the Iberian Peninsula exhibits high values of favourability to host this African species for the current climatic conditions. Furthermore, future forecasts indicated an increase in favourability for this area. The highly favourable areas we detected in the south of the Iberian Peninsula are already regularly receiving individuals of the species. These observations are very likely vagrant birds dispersing from recently colonised breeding areas in northern Morocco, which may indicate a continuous process of colonisation towards the north, as has occurred during the last decades in Northern Africa.

**Conclusions:**

We cannot anticipate when the House Bunting will establish on the European continent because colonisation processes are usually slow but, according to our results, we predict its establishment in the near future. We have also identified those areas hosting favourable conditions for the species in Europe. These areas are a potential focal point for the colonisation of this and other African birds if the climate continues to warm.

## Background

The distribution of species, both in space and time, is affected by environmental, biological, historical and anthropogenic factors [1]. According to several studies, however, climate appears to be the most relevant factor for several taxa at large scales [2–5]. In recent decades, it has been demonstrated that the global climate is becoming warmer [6], with widespread effects on biological systems [7–10]. This recent climate change has already affected the distribution of many species [11–14] and is modifying the margins of their distributions over short periods of time [15–17]. This process is more notorious in vagile species such as birds, which are frequently monitored and included in citizen science platforms [18, 19].

Modifications in birds’ distribution [20, 21] and phenology [22–24], as a response to a warmer climate, constitute an interesting line of work that has begun to be addressed in recent decades. Predicting range shifts is a current challenge for biogeography because bird ranges are expected to move in response to temperature, precipitation and habitat changes [13, 25, 26]. Latitudinal and elevational movements [20, 27, 28] are relevant both for the species that suffer the changes and for the other species that reside in the same areas, as community compositions are ultimately altered [14]. This adds uncertainty to the future status of natural populations and communities, and forces conservation programmes to be adapted to the new species distributions [29, 30].

This topic is of particular relevance in southern Spain and northern Morocco, where an important biogeographical barrier separates the North African and the southern European fauna and flora. The Strait of Gibraltar is a bridge for many migratory species [31, 32] and an effective biogeographic barrier for many other taxa [33–35]. This area delimits the northern distribution of some African bird species, such as the House Bunting (*Emberiza sahari*), the Moussier’s Redstart (*Phoenicurus moussieri*) or the Black-crowned Tchagra (*Tchagra senegalus*) [36]. If they overcome this barrier to move further north, they cross into Europe, where they come into contact with different species and, in some cases, new habitats, as is currently happening with some typically African birds [37–41]. In recent decades, the Iberian Peninsula has been successfully colonised by a number of species of African birds, including the Black-shouldered Kite (*Elanus caeruleus*) [42], the White-rumped Swift (*Apus caffer*) [43], the Little Swift (*A. affinis*) [44] and, more recently, the African Long-legged Buzzard (*Buteo rufinus cirtensis*) [45] and the Common Bulbul (*Pycnonotus barbatus*) [46]. In addition, there are other species that, although not yet established as breeders, are regularly observed as a process of the unexceptional arrival of individuals. Some examples of these are Rüppell’s Vulture (*Gyps rueppellii*) [47], the African White-backed Vulture (*G. africanus*) [2], the Lanner Falcon (*Falco biarmicus*) [48], Moussier’s Redstart [49] and the House Bunting [49]. Mostly due to the relative proximity between Africa and Europe in the western Mediterranean, the southern part of the Iberian Peninsula is an adequate place for the potential establishment of these species, acting as a focal point in the colonisation process of Europe by African birds [2, 45, 47].

In recent decades, the House Bunting has increased its distribution range towards the north in Morocco until it has reached the Mediterranean coast [50]. For this reason, we could expect it to colonise south-western Europe if the environmental conditions are adequate, reaching the Iberian Peninsula. The aim of this study is to determine if the climatic conditions of southern Europe—both current conditions and several climate change estimates based on different emission scenarios —are suitable to host the House Bunting as a breeder, and also to identify where these conditions exist. This information will be of interest in monitoring the expansion of African species as a result of climate change.

## Results

**Fig. 1:**
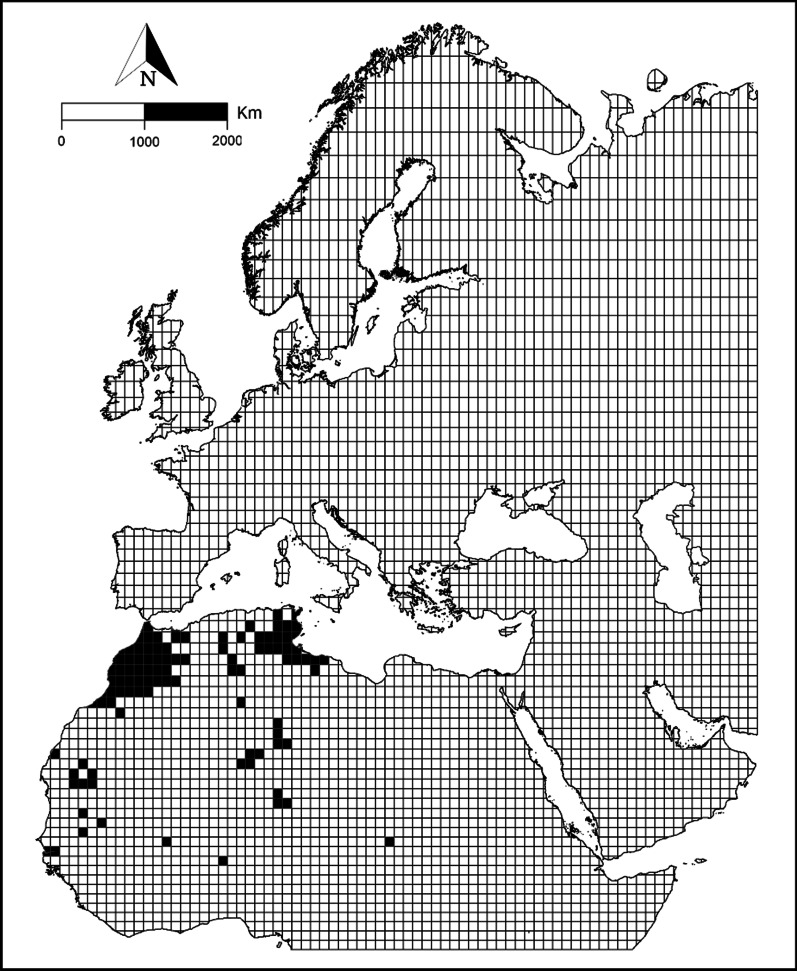
Current House Bunting breeding presences in the study area (shown in black), which was divided in grid cells of 1-degree latitude × 1-degree longitude. These presences were the only ones used in the modelling process. Map created using ArcMap software (ArcGIS 10.4.1) https://desktop.arcgis.com/es/arcmap/

Our search for data from eBird resulted in 5520 records throughout this century, until the end of 2020. With these records we obtained a binary target variable representing breeding/not breeding at each Operational Geographic Unit (OGU) (breeding: *n*_*1*_ = 104, not breeding: *n*_*0*_ = 4073). All OGUs where the species breeds were in Africa (Fig. 1). The prevalence of the species in the study area was 0.025.

**Fig. 2:**
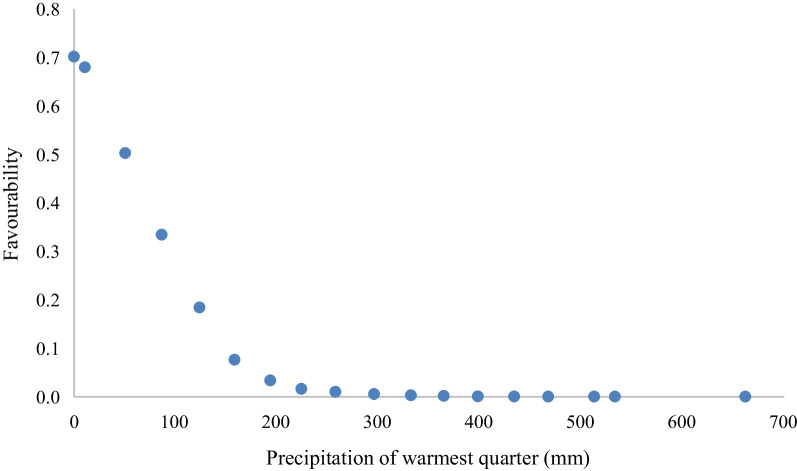
Sensitivity of favourability function to the variable “Precipitation of the warmest quarter”. The variable’s range values were divided into 20 points and each point represents the mean favourability value

The mathematical model included three variables, two of them related to climate, whose future values were within the range of values for the present in more than 99.8% of grid cells, and altitude (Table 1). Dry weather low in precipitation in the warmest quarter was the best predictor for House Bunting breeding because this variable was the first to be entered in the stepwise procedure and, under the Wald test, this variable possessed the greatest weight in the model. According to the sign of the variables’ coefficients in the logit function, precipitation in the warmest quarter had a negative effect on the species distribution while annual precipitation and altitude had a positive effect (Table 1). The increase in precipitation of the warmest quarter in the range of 0–200 mm negatively affected the favourability for the House Bunting breeding, and precipitation values above 200 mm always resulted in negligible favourability (Fig. 2).

**Table 1:** Variables entered into the logistic regression model via a forward–backward stepwise selection process, ranked by their order of entrance

Variable	β	S.E.	Wald	*p*
Precipitation of warmest quarter	− 0.0261	0.00406	41.345	< 0.001
Annual precipitation	0.00112	0.000404	7.689	0.006
Altitude	0.000503	0.000206	5.964	0.015
Constant	− 3.020	0.175	298.267	< 0.001

βs are the coefficients in the logit function, S.E. is the standard error of these coefficients, Wald is the Wald’s statistics value (representing the relative importance of the variable in the model) and *p* is the significance of the coefficients according to the Wald test

**Fig. 3:**
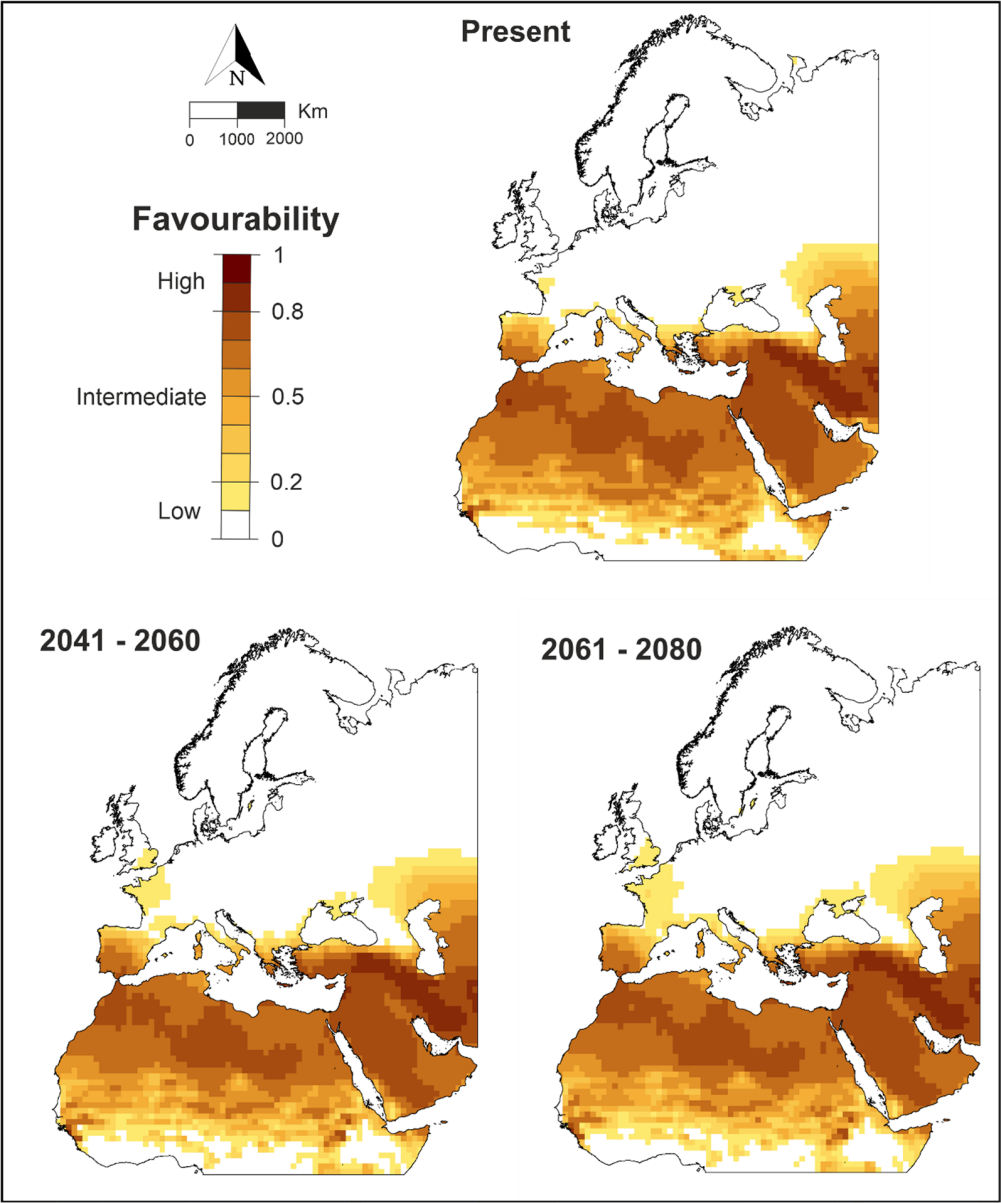
Cartographic representation of the current climatic favourability for House Bunting breeding in each operational geographic unit of the study area and the ensemble climatic favourability models for future periods of time. Maps created using ArcMap software (ArcGIS 10.4.1) https://desktop.arcgis.com/es/arcmap/

The southwestern quadrant of the Iberian Peninsula showed high values of favourability to host the House Bunting, both for the current climatic conditions and for the different scenarios of future climate change (Fig. 3). When focusing on the southernmost areas, the Iberian Peninsula was found to be as favourable as Moroccan native areas. Considering the map for the near future (2041–2060), an increase in favourability was detected in the south of the Iberian Peninsula, especially in the area of the Strait of Gibraltar and the province of Malaga, and the favourability values increased in France and the south of the United Kingdom, although these values were always lower than 0.2. Favourability continued to increase slightly in the Iberian Peninsula, France and the south of the United Kingdom according to forecasts for the distant future (2061–2080), but no major changes were expected in relation to the near future.

**Fig. 4:**
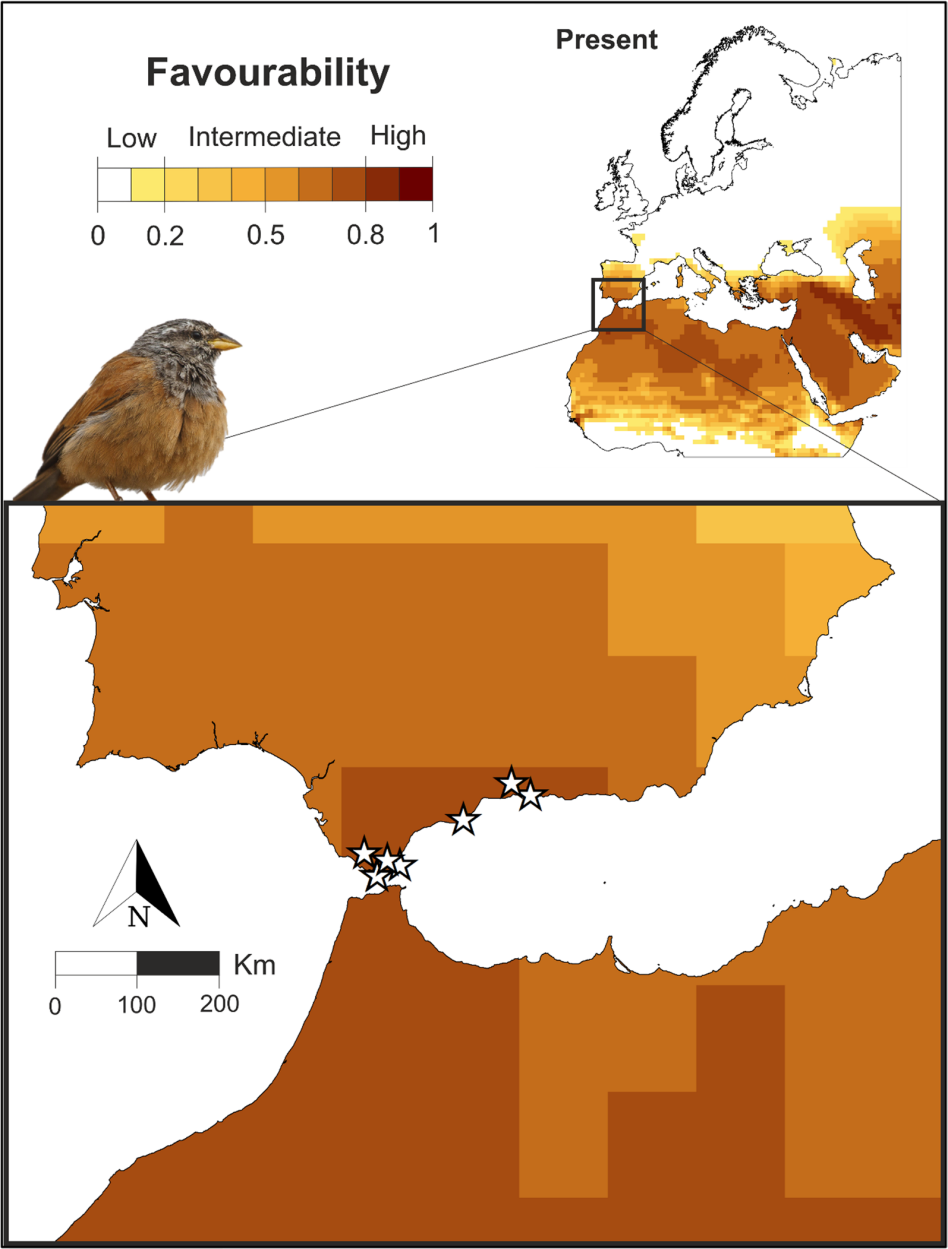
Current climatic favourability for the breeding of the House Bunting in the south of the Iberian Peninsula and northern Morocco. The stars represent confirmed records of the species in Europe. Maps created using ArcMap software (ArcGIS 10.4.1) https://desktop.arcgis.com/es/arcmap/

The discrimination capacity of the model was high (AUC = 0.811) and highly significant (*p* = 1.782 × 10^–27^). According to the classification accuracy, the model showed higher sensitivity (0.923) than specificity (0.595), a low under-prediction rate (UPR = 0.003) and a high over-prediction rate (OPR = 0.945). At the time of this study, the only records of the House Bunting in Europe were found in the south of the Iberian Peninsula, in areas that our model detected as the most favourable for the species (Fig. 4). The first House Bunting was observed in Europe on the 5th of August 2009, in Tarifa city (Cadiz) and the second was observed on the 13th of October of the same year in Nerja (Malaga). In recent years, regular arrivals of single individuals have been detected: in 2018 in Frigiliana (Malaga), in 2019 in Tarifa (Cadiz), in 2020 in Puertollano (Cadiz), in 2021 in both Gibraltar and Algeciras (Cadiz), and in 2022 in Fuengirola (Malaga). All these records of the species in the Iberian Peninsula took place during the dispersal period, just after the breeding season.

**Fig. 5:**
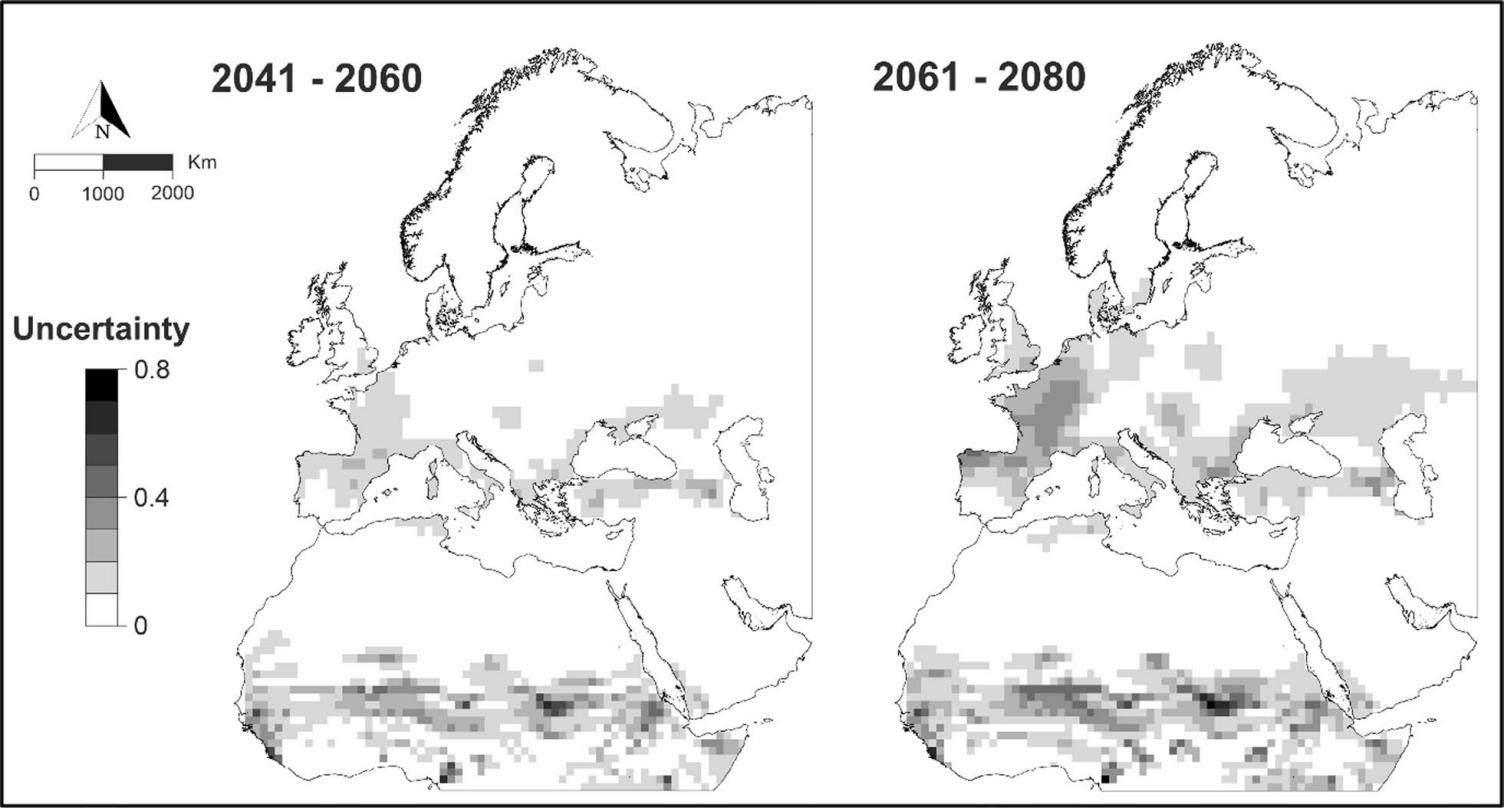
Climatic uncertainty associated with different climate change scenarios analysed for two different future time periods. Maps created using ArcMap software (ArcGIS 10.4.1) https://desktop.arcgis.com/es/arcmap/

There was a low mean uncertainty of less than 0.1 associated with future climate scenarios: 0.065 for the period 2041–2060 and 0.084 for the period 2061–2080 (Fig. 5). For the distant future, there was an increase in uncertainty values in some European areas, including the northern half of the Iberian Peninsula, France, Belgium, the Netherlands, northern Italy, Bulgaria, Hungary and Slovakia, although in the near future the uncertainty values were much lower. The highest uncertainty values were in West Africa, Chad and Sudan, particularly for the period 2061–2080, whereas low uncertainty values were found in the south of the Iberian Peninsula and North Africa.

## Discussion

Over the last six decades, the House Bunting has colonised approximately 300 kms to the north in Morocco, reaching the coastal areas of North Africa [51]. By the beginning of the twenty-first century, it had moved to the east, colonising as far as the frontier between Morocco and Algeria [50]. The next step could be to colonise Western Europe due to the proximity of this area to the current populations of the species recently established in Northern Africa. Our distribution models showed that southern Europe, specifically the southwestern quadrant of the Iberian Peninsula, hosts adequate environmental conditions for the establishment of the House Bunting, both based on the current climatic conditions and for different scenarios of future climate change.

Two of the three significant predictive variables included in the favourability model were associated with precipitation. In addition, the only non-climatic variable, altitude, was the last to be included in the model, suggesting a finer-scale effect [52]. Consequently, the House Bunting’s breeding distribution is characterised by scarce precipitation during the summer, which makes sense because it is a species that occupies arid areas. Climate change is expected to decrease the annual mean precipitation in many mid-latitude regions, especially in the Mediterranean basin [53]. It is also expected to increase both day- and night-time temperatures and reduce the number of cold nights, as well as increasing the frequency and duration of heat waves [53], which will result in a warmer and drier environment in the European region, mainly in the south. Under these conditions, the possibility of African bird species becoming established in southern Europe will likely increase in coming years.

Our results showed that in Europe, the south of the Iberian Peninsula is the area with the best climatic conditions to host the House Bunting. Furthermore, it is the region closest to current and expanding populations of the species in northern Africa. The areas recently colonised in northern Morocco, where the species has occupied mostly urban areas and is already nesting [51], showed the same values of favourability as those of the southernmost tip of the Iberian Peninsula, which is as close as 14 km at the shortest distance, but does possess the natural barrier of the Strait of Gibraltar. In addition, the south of the Iberian Peninsula and North Africa had some of the lowest uncertainty values, indicating that our results were more consistent in these areas. The forecasts for the period 2061–2080 had the highest uncertainty, which is normal when the forecast time period is further away from the present [53]. Regardless, it is expected that if the species continues its northward spread, it will establish itself in some of these favourable areas in the near future. In addition, according to our results, future forecasts indicate that the favourability will increase in these areas. Furthermore, our model showed numerous OGUs favourable for the species in southern Europe (especially in the southern half of the Iberian Peninsula) where breeding has not yet been reported. For this reason, it showed a low specificity and a high over-prediction rate. In addition, it showed a high sensitivity and a low under-prediction rate as more than 90% of OGUs with reported breeding were classified as favourable for the species. However, apart from climate other driving factor of species distribution, such as urban expansion, may be influential in the establishment of the House Bunting in southern Europe [4, 54], given that the species prefers urban areas in Northern Africa and most of the eBird records in Spain are in towns and cities.

The favourable areas we detected in the south of the Iberian Peninsula are already regularly receiving individuals of the species. To date, these individuals have been detected in this area during the dispersal period of the species. In this way, the model can be validated because all records in Europe were included in those areas that were, previously and with independent data, predicted as highly favourable. These observations correspond very likely with vagrant birds dispersing from the recently colonised breeding areas in northern Morocco, which may indicate a continuity in the process of colonisation towards the north if they can surpass the sea barrier. There are recent cases of other African birds that have already experienced a similar expansion pattern, colonising the Iberian Peninsula, such as the Common Bulbul [46] or the Cream-colored Courser (*Cursorius cursor*) [55].

If the House Bunting becomes established in the south of the Iberian Peninsula, it will likely begin to colonise other areas of the European continent, as it will be much easier once the important biogeographical barrier of the Strait of Gibraltar has been overcome. These areas will likely be located in the southwestern quadrant of the Iberian Peninsula because, according to our results, they have the greatest favourability values. The species could then use these favourable areas as steppingstones to expand into the rest of Europe. This has previously occurred with the Black-shouldered Kite, which started breeding in Extremadura and Salamanca (central west Spain) in 1975, then moved northwards and became established in France in 2013 [56–58]. Furthermore, the establishment of the House Bunting in Europe would imply a change in communities with similar species, such as the Rock Bunting (*Emberiza cia*). Some of these changes in communities could be hybridisation, which have already occurred with other African bird species established on the European continent. Hybridisation between the African Long-legged Buzzard and the Common Buzzard (*B. buteo buteo*) has been detected in Pantelleria Island (Italy) [59] and the Strait of Gibraltar [60]. Other changes could be related to competition or changes in trophic structures [61].

There are several factors that could facilitate the establishment of the House Bunting in the south of the Iberian Peninsula. The first is the short distance between the already occupied areas in northern Morocco and the favourable unoccupied areas in southern Spain, which could facilitate the natural arrival of individuals. The second is the fact that the House Bunting usually occupies urban environments [62], which may imply some advantages such as greater availability of resources, less competition or protection against predators [63, 64]. Thirdly, the presence of large ports and very intense maritime traffic between cities in Morocco, Spain and Gibraltar may increase the number of individuals that arrive in these areas [50]. It should be noted, however, that individuals also arrive naturally, as there are records of House Buntings in places where there are no ports, such as Fuengirola or Frigiliana.

The possibility does exist that the House Bunting will not eventually become established on the European continent and its distribution limit will be restricted to North Africa. In this case, it is worth highlighting the important biogeographical barrier that the Strait of Gibraltar represents [33, 34]. We expect, however, that the species will eventually be able to colonise the south of the Iberian Peninsula, as has already happened with other African bird species [43–46]. For this reason, citizen science platforms, species monitoring and follow-up programmes will play important roles in continuing to study the evolution of the species in Europe and in validating the favourability models presented in this study.

## Conclusions

We cannot anticipate when the House Bunting will establish on the European continent because colonisation processes are usually slow [65]. Nevertheless, considering the fast northward expansion that the species has undergone in recent years until reaching northern Morocco, the fact that the south of the Iberian Peninsula has suitable climatic conditions for it, and the relative regularity of individuals that are already arriving in the south of the Iberian Peninsula, we predict the colonisation of the species in the near future. In this study, we have focused on a single species but these results can be extrapolated to other African birds, with the possibility of an Africanisation of the European fauna.

## Methods

### Study area

The study area comprised the land region from 20° 00′ W to 60° 00′ E, and from 09° 30′ N to 70° 00′ N, thus covering the Western Palearctic and surrounding areas (Fig. 1). We considered the entire Western Palearctic because it is a relevant biogeographic unit for studying the potential expansion of the House Bunting and other bird species. This study is part of a broader project (LifeWatch ERIC, EnBiC2-Lab) that is focused on an intercontinental scale to study the effects of climate change on species distributions. This area has a high climatic heterogeneity, involving sub-tropical, desert, Mediterranean, Atlantic and tundra climates [66, 67], and fully covers the current breeding territories of the House Bunting.

The study area was divided using a grid cell of 1-degree latitude × 1-degree longitude to obtain operational geographic units (OGUs, *n* = 4177), using the *Create Fishnet* and *Intersect* tools from ArcGIS software.

### The species

The House Bunting is closely associated with urban environments [62]. It ranges from the Maghreb to Chad and Mauritania in Northern Africa. Formerly, it lived only in desert or semi-desert areas, breeding in very few localities north of the Atlas Mountains before 1950. Since the mid-twentieth century, however, the species has shown a very clear tendency to increase its distribution range towards the north in Morocco, Algeria and Tunisia, occupying exclusively urban habitats. Thévenot et al*.* [51] described in detail the House Bunting’s expansion towards the north in Morocco, where it was detected in Casablanca and Guercif in the 1960s. In the 1980s, it had reached Oujda, Rabat, Fez and Meknes; by 1995 it was located in Kenitra, Sidi Slimane and Sidi Kacem, just 180 kms from the Strait of Gibraltar; and at the beginning of the twenty-first century, it had already reached Tangier. In recent years, the House Bunting has colonised large areas of northern Morocco including the Mediterranean coast [50]. Currently, single individuals of the species are beginning to be observed regularly in the south of the Iberian Peninsula, with specific observations in the provinces of Cadiz and Malaga (see Fig. 4).

### Species distribution data

In the last decade, citizen science platforms have become key sources for obtaining global biodiversity data for certain taxa, such as birds [68]. For this study, distribution data to determine the breeding area of the House Bunting was obtained from *e-Bird* (https://ebird.org/home), an international citizen science platform specialising in birds, with hundreds of thousands users around the world. We used eBird records of the current century until the end of 2020 that were within the breeding season of the House Bunting (from late February to late July) [69]. From these records, we identified the OGUs where breeding of the species was assumed, and then used only these African presences to model the distribution of the species in the study area.

Recent reports of the species in Europe (Fig. 4) were obtained from *e-Bird* (https://ebird.org/home) and the SEO/BirdLife Rarities Committee [70, 71]. All these records, which were confirmed with the help of pictures of each observation of the species, were used to validate the results provided by the distribution model.

### Predictor variables and future scenarios

In the biogeographic modelling procedure, we used a set of 21 environmental variables (Table 2), two of them related to topography and the remaining 19 related to climate between 1950 and 2000. We included topography together with climate in the modelling approach because otherwise their differentiated influences may be confounded and mistakenly attributed only to climate [4]. Furthermore, it is important to balance the impact of climate change against the inertia promoted by other influential factors that will not change in the future, such as topography, when forecasting species distribution [72, 73]. These variables were digitised in raster format at a resolution of 1 km^2^ pixels. Values of these variables at each OGU were obtained by averaging the values of the 1 km^2^ pixels within them using the ZONAL function of ArcGIS 10.4.1 software [74].

**Table 2:** Variables selected to model the breeding distribution of the House Bunting, grouped by environmental factor

Code	Variable	Units	Source
*Topography*			
Alti	Altitude	m	(1)
Slope	Slope	Degrees	(2)
*Climate*			
Tmean	Annual mean temperature	°C	(3)
Rtday	Mean diurnal temperature range	°C	(3)
Isot	Isothermally	%	(3)
Season	Temperature seasonality	Standard deviation	(3)
Tmax	Maximum temperature of warmest month	°C	(3)
Tmin	Minimum temperature of coldest month	°C	(3)
Rtan	Temperature annual range	°C	(3)
Twet	Mean temperature of wettest quarter	°C	(3)
Tdry	Mean temperature of driest quarter	°C	(3)
Twarm	Mean temperature of warmest quarter	°C	(3)
Tcold	Mean temperature of coldest quarter	°C	(3)
Prec	Annual precipitation	mm	(3)
Pmax	Precipitation of wettest month	mm	(3)
Pmin	Precipitation of driest month	mm	(3)
Cvp	Precipitation seasonality	Coefficient of variation	(3)
Pwet	Precipitation of wettest quarter	mm	(3)
Pdry	Precipitation of driest quarter	mm	(3)
Pwarm	Precipitation of warmest quarter	mm	(3)
Pcold	Precipitation of coldest quarter	mm	(3)

Data sources: (1) US Geological Survey [75]; (2) calculated from *Alti* with ArcGIS software; (3) Hijmans et al*.* [76]

Expected future values of the climatic variables were obtained for the periods 2041–2060 and 2061–2080 (https://worldclim.org/). In the same way as in Chamorro et al*.* [74], four different Representative Concentration Pathways (RCPs) were used to project future CO_2_ emissions: 2.6, 4.5, 6.0 and 8.5 [77]. Two different Global Circulation Models (GCMs) were also used: HadGEM2-ES and NorESM1-M [29, 78]. We used these two GCMs because they have been found to be good predictors of future climate in both Europe and Africa [79]. This process resulted in eight sets of expected values of the climatic variables for each period of time. We assessed whether the climatic variables that entered the model were within the range of values that these variables had for the present.

### Model for the present

First, Spearman correlation coefficients (*r*) were calculated between the selected environmental variables to reduce multicollinearity. For each pair of variables with *r* > 0.8, only the variable with the highest individual predictive power was retained [80, 81]. In species distribution models in which many variables can potentially predict the presence or absence of a species, some variables may be incorporated by chance [82]. This type of error (type I error) can be controlled by evaluating the *False Discovery Rate* (FDR), as proposed by Benjamini and Hochberg [83]. Based on the set of pre-selected variables in the previous step, only the variables whose significance in the score test was less than an FDR value of 0.05, were accepted in subsequent modelling procedures [84].

Next, a comprehensive model for the current probability of breeding at every OGU according to its climatic conditions was obtained by multivariate forward–backward stepwise logistic regression. This procedure began with a null model (model with no predictor variables) that produced a constant probability of breeding at each OGU equal to the prevalence (OGUs where species have been confirmed to breed in relation to the total number of OGUs). In the first step, the procedure selected the variable with the most significant relationship to the distribution of the species, according to the Rao’s score test. In the following steps, the variable most significantly related to the residues not explained in the previous step was added to the model, until the step in which any variable significantly increased the predictive capacity of the model was reached [85]. By using a forward–backward stepwise variable selection procedure, before adding a new variable to the model, the possibility of improving its predictive capacity was evaluated by eliminating any of the variables introduced in a previous step. Finally, a significant combination of predictors was obtained (*y* or *logit*), where the coefficients of the predictor variables were estimated using a machine learning algorithm based on a likelihood ascent gradient. The relative weight of each variable in the final model was assessed using the Wald test [86].

To eliminate the effect of prevalence on the probability values obtained and to attain values that depend exclusively on the response of the species to environmental variables [87], favourability values (*F*) were obtained by applying the *Favourability Function* [88], using the following Eq. (1):

1 $$F = 1 - \frac{1}{{1 + e^{{\left( {\ln \frac{P}{1 - P} - \ln \frac{n1}{{n0}}} \right)}} }}$$

where *P* is the probability value obtained through logistic regressions, *e* is the Euler’s number, *ln* is the natural logarithm, and *n*_*1*_ and *n*_*0*_ are the number of OGUs where breeding was reported or not reported, respectively.

The favourability refers to the degree, from 0 (minimum favourability) to 1 (maximum favourability), to which the environmental conditions favour the breeding of the species [89, 90], with *F* = 0.5 being the threshold separating favourable from unfavourable areas. A local favourability value of 0.5 indicates that the local probability of the species breeding is the same as its prevalence in the study area, meaning the probability expected by a null model unaffected by environmental predictors, where breeding is neither favoured nor unfavoured by the environment [88]. Those areas with *F* > 0.5 therefore favour the species breeding, whereas *F* < 0.5 indicates areas with conditions that disfavour breeding. Nevertheless, the use of a favourability value of 0.5 as a cut-off point for crisply distinguishing favourable from unfavourable areas is not sufficiently informative [91] due to the continuous and fuzzy character of favourability [89]. For this reason, we opened a gap between the values considered as clearly favourable and clearly unfavourable. Each OGU was classified into three categories, depending on their favourability values. If the predicted favourability was higher than 0.8, which means that the odds are more than 4:1 favourable to the species, the OGU was considered as highly favourable; those areas with favourability values lower than 0.2 (odds less than 1:4) were considered unfavourable to the species; and the remaining areas were considered to be of intermediate favourability (0.2 ≤ *F* ≤ 0.8) [74, 92]. All modelling processes were run with the IBM SPSS Statistics 25 software package.

### Model assessment

The discrimination capacity of the resulting model was evaluated using the Area Under the Receiver Operating Characteristic (ROC) Curve, known as the AUC [93, 94], which has an associated significance value. As described by Fielding and Bell [95], Muñoz and Real [92] and Barbosa et al*.* [96], we applied a set of classification measures, whose values range from 0 to 1. These measures were sensitivity (the conditional probability of OGUs with reported breeding being classified as favourable), specificity (the conditional probability of OGUs with no reported breeding being classified as unfavourable), the over-prediction rate (OPR: the proportion of OGUs with no reported breeding in the area with favourability higher than 0.5) and the under-prediction rate (UPR: the proportion of OGUs with reported breeding in the area with favourability lower than 0.5).

### Projection to future climatic scenarios

We obtained future climatic favourability values (*F*_*f*_) by replacing the present values of the climatic variables in the *logit* (*y*) of the favourability equation [88] with the expected future values according to each RCP and GCM, and for each future period of time [29, 72, 97]. This process resulted in eight expected climatic favourability models for each period. An ensemble forecasting of the models was obtained for each period of time by calculating the mean values of the eight future climatic favourability models at each OGU.

The uncertainty of the ensemble forecasting was computed using fuzzy set theory [98], given that favourability values may be considered to be the degree of membership in the fuzzy set of areas favourable for House Bunting breeding. Thus, the favourability function is the membership function that assigns each OGU their degree of membership value [88]. The uncertainty was computed as the difference at each OGU between the fuzzy union of the eight models (the maximum value of favourability of any of them at the OGU) and their fuzzy intersection (the minimum value of favourability of any of them at the OGU) [99].

## Data Availability

The data that support the findings of this study are available in Dryad at https://datadryad.org/stash/share/TgrQQ7-7ZBhuncU7gdFkeZ6lTXYEFm-zZXVbGDjS0x8, https://doi.org/10.5061/dryad.bzkh189fb. These data were derived from the platform eBird available in the public domain: https://ebird.org
